# Risk prediction of pneumocystis jirovecii pneumonia in kidney transplant recipients using a clinical nomogram

**DOI:** 10.3389/fcimb.2026.1778937

**Published:** 2026-05-13

**Authors:** Qu Liu, Zhenghui Wang, Yujiao Song, Lanlan Liu, Shuang Qiu, Xinxin Niu, Jie Liu, Hailong Jin, Qing Zhang

**Affiliations:** 1 Department of Organ Transplantation, The Third Medical Center of Chinese People's Liberation Army (PLA) General Hospital, Beijing, China; 2 Faculty of Hepatopancreatobiliary Surgery, The First Medical Center of Chinese People’s Liberation Army (PLA) General Hospital, Beijing, China; 3 Laboratory Department, The Third Medical Center of Chinese People's Liberation Army (PLA) General Hospital, Beijing, China; 4 Oncology Clinical Development, Johnson & Johnson (J&J) Innovative Medicine Research & Development, Beijing, China

**Keywords:** kidney transplantation, nomogram, pneumocystis jirovecii pneumonia, prediction, risk factors

## Abstract

**Background:**

Pneumocystis jirovecii pneumonia (PJP) remains a life-threatening opportunistic infection in renal transplant recipients despite universal prophylaxis, with its risk factors in this setting poorly defined. This study aimed to investigate these factors and develop a nomogram to predict PJP risk, identifying patients requiring extended prophylaxis.

**Methods:**

Demographic characteristics and perioperative outcomes of consecutive patients who underwent kidney transplantation from DCD or DCBD donors at a high-volume kidney transplant center in China between January 2016 and January 2019 were retrospectively analyzed. A nomogram was developed using data from a retrospective training cohort based on a Cox regression model. The model was tested in a validation cohort.

**Results:**

Among 702 patients, 52 developed postoperative PJP (7.4%). PJP occurred in 38 of 491 patients (7.73%) in the training cohort and in 14 of 211 patients (6.64%) in the validation cohort. The nomogram included the following variables: postoperative CD4+T cell count, CD8/lymphocyte ratio (CD8/L), blood glucose, NEU percentage and CRP ≥ 8μg/mL. The C-statistics and AUC values of the nomogram for PJP prediction were 0.88, 0.93, and 0.89 in the training, validation, and entire cohorts, respectively. The incidence of PJP was significantly higher in the high-risk group than in the low-risk group across all three cohorts (*p* < 0.001).

**Conclusions:**

Renal transplant recipients with lower CD4+T cell counts and higher CD8/L, blood glucose, NEU percentage, and CRP levels are at increased risk of PJP. The nomogram may serve as a useful and practical tool for predicting the individualized risk of PJP after kidney transplantation, facilitating the identification of high-risk patients requiring extended prophylaxis.

## 1 Introduction

Pneumocystis jirovecii pneumonia (PJP) is one of the most common opportunistic fungal infections and frequently occurs in immunocompromised individuals (Eitner et al., 2011; Goto and Oka, 2011; Cheng et al., 2022). Patients who undergo kidney transplantation are particularly susceptible to PJP, which can occur at almost any stage after transplantation, with a peak incidence during the first 6 months (Yetmar et al., 2023). In transplant recipients, the incidence of PJP has been reported to range from 5% to 24% in the absence of routine prophylaxis (Goto and Oka, 2011; Garg et al., 2018; Saadatzadeh et al., 2024), with high mortality reaching 50% (Neff et al., 2009; Nseir et al., 2024), posing a serious threat to recipient and graft survival (Zou et al., 2022b).

Pneumocystis jirovecii can readily cause outbreaks among renal transplant recipients receiving excessive immunosuppressive therapy (Fritzsche et al., 2013; Szydłowicz et al., 2019; Permpalung et al., 2021). Previous reports have shown that an increased risk of PJP infection is associated with various factors, including treatment for acute rejection, use of high-dose corticosteroids, and cytomegalovirus (CMV) infection (Lee et al., 2017, 2022; Zou et al., 2022a). Recent studies have also shown that azathioprine (AZA) treatment, induction therapy (including rituximab-containing regimens), lymphopenia, older age, ABO-incompatibility, and decreased eGFR are risk factors for PJP infection in renal transplant recipients (Cheng et al., 2024; Saadatzadeh et al., 2024; Wang et al., 2026). Prophylaxis with trimethoprim-sulfamethoxazole (TMP-SMX) can effectively reduce the incidence of PJP during the post-transplant period (Chapman et al., 2019; Fishman and Gans, 2019; Chantapoh et al., 2025). The recommended duration of PJP prophylaxis varies: European guidelines suggest 4 months, the Kidney Disease: Improving Global Outcomes (KDIGO) guidelines recommend 3–6 months, and the American Society of Transplantation (AST) advises a longer period of 6–12 months (Goto and Oka, 2011; Fishman and Gans, 2019). Notably, 0.4–2.6% of PJP cases occurred post-transplantation despite prophylaxis (Neff, 2009; Saadatzadeh et al., 2024; Konwai S et al., 2026), whereas Moghaddam et al. reported that among 209 SOT recipients (198 kidney transplants), no patient developed breakthrough infection (Hosseini-Moghaddam et al., 2019). Late-onset PJP remains a concern in the era of universal prophylaxis after transplantation (Mulpuru et al., 2016; Lee et al., 2022). However, the risk factors for PJP in renal transplant recipients receiving prophylaxis have not been fully defined, and no consensus exists regarding which high-risk individuals may benefit from prolonged or re-initiated prophylaxis. To date, the roles of indicators reflecting immunocompromised status, inflammation, and metabolic abnormalities in predicting PJP risk remain unclear. Thus, current risk assessment models based on immunosuppressive therapy and CMV infection appear to have limitations and urgently need to incorporate more comprehensive risk factors to identify high-risk individuals. Moreover, a validated predictive model for accurately estimating PJP risk is currently lacking.

Nomograms are graphical models that enable calculation of the overall probability of a specific clinical outcome for an individual patient based on the combined effects of multiple risk factors (Feng et al., 2022; Obi and Omata, 2024; Zeng et al., 2025). This method offers more advantages than traditional analytical methods. Many nomograms have been used as predictive tools for various diseases (Zheng et al., 2025). Therefore, it is necessary to further explore the nomogram approach to develop a novel risk prediction model for PJP in renal transplant recipients.

In this study, based on a large training cohort of renal transplant recipients under prophylaxis, we aimed to investigate the risk factors for PJP by analyzing pre- and post-operative clinical data and to build an effective nomogram model to predict the risk of PJP in patients who may be benefit for prolonged prophylaxis. The model is designed to stratify patients by risk, thereby helping identify high-risk patients and guide individualized preventive strategies and post-transplant management.

## 2 Materials and methods

Patients who received kidney transplants from donation after cardiac death (DCD), donation after brain plus cardiac death (DCBD), or living donors at a high-volume kidney transplant center in China between January 2016 and November 2019 were included. This case-control study was approved by the Ethics Committee of the Chinese PLA General Hospital. Written informed consent was obtained from all enrolled patients. This retrospective cohort study was registered with ResearchRegitry.com (Unique Identification Number: research registry8604). The study was conducted in accordance with the STROCSS criteria (Messiaen et al., 2017). The inclusion criteria were as follows: (1) undergoing kidney transplantation, (2) HIV-negative status, and (3) maintenance immunosuppressive therapy with calcineurin inhibitors (CNI) combined with mycophenolate mofetil (MMF) and glucocorticoids. Patients were excluded for the following reasons: (1) age <18 years, (2) renal transplant recipients undergoing other organ transplantations, (3) incomplete preoperative baseline or postoperative data, and (4) loss to follow-up after kidney transplantation.

### 2.1 Patient variables and definitions

Baseline demographic data, as well as perioperative and pathological outcomes, were retrospectively obtained from the Chinese Scientific Registry of Kidney Transplantation (https://www.csrkt.org.cn/) and medical records. For both cases and controls, data were collected on the following variables: recipient age, sex, BMI, blood type, dialysis modality, induction therapy, glucocorticoid and immunosuppressive drugs, clinical symptoms, lung CT characteristics, laboratory test results, occurrence of delayed graft function (defined as the need for hemodialysis during the first week after transplantation), occurrence of cytomegalovirus (CMV) infection, prophylaxis for PJP and CMV, and clinical diagnosis. Pretransplant evaluation data also included viral serologic screening for human immunodeficiency virus (HIV), CMV, and hepatitis B and C viruses. The following blood lymphocyte subsets were routinely assessed: CD4+T cell count (CD4), CD8+T cell count (CD8), CD4+/lymphocyte ratio (CD4/L), CD8+/lymphocyte ratio (CD8/L), and CD4/CD8 ratio. Laboratory data for both groups were collected at the second month after transplantation during the maintenance therapy phase.

The diagnosis of PJP was confirmed according to the Guidelines of the AST Infectious Diseases Community of Practice [14] and was mainly based on the following criteria: (1) clinical symptoms consistent with PJP, including fever, cough, dyspnea, and hypoxemia; (2) chest CT findings generally showing diffuse interstitial involvement, such as ground-glass opacities and reticular opacities; (3) microbiological diagnosis based on positive P. jirovecii PCR or direct immunofluorescence results in induced sputum or bronchoalveolar lavage fluid (BALF), combined with metagenomic next-generation sequencing (mNGS) for suspected cases; and (4) a favorable response to anti-PJP therapy. In this study, all patients with PJP underwent chest computed tomography scanning.

CMV infection was defined as a CMV DNA PCR titer≥500 copies/mL in whole blood, accompanied by clinical symptoms and signs of CMV infection after kidney transplantation.

### 2.2 Immunosuppressive regimen

In both cases and controls, induction therapy for renal transplant recipients consisted of basiliximab, anti-thymocyte globulin, or anti-CD25 monoclonal antibodies, and perioperative immunosuppressive therapy consisted of a CNI (tacrolimus or cyclosporine A), MMF, and glucocorticoids. The total glucocorticoid dose administered during surgery was 500–1000 mg. The maintenance immunosuppressive regimen consisted of CNI (tacrolimus or cyclosporine A), mycophenolate mofetil (MMF), and glucocorticoids.

### 2.3 Prophylaxis for PJP and CMV

PJP prophylaxis was routinely administered with oral TMP-SMX (80/400 mg) once daily or three times weekly for 3–4 months after transplantation, based on renal function and clinical assessment. In patients with intolerance or contraindications to sulfa drugs, a second-line agent (dapsone 100 mg once daily) was used for prophylaxis. CMV prophylaxis with oral valganciclovir was administered for more than 3 months after transplantation.

### 2.4 Statistical analysis

Continuous variables were presented as mean ± SD or median and interquartile range (IQR), as appropriate. The Mann-Whitney U test was used to compare continuous variables. Categorical variables were compared between groups using the chi-square test or Fisher’s exact test, as appropriate. Patients were randomly allocated to the training and validation cohorts using a split-sample randomization method at a ratio of 7:3 (training: validation = 491:211). Univariate and multivariate logistic regression analyzes were performed to identify independent risk factors for PJP in the training cohort. The nomograms were constructed by converting each regression coefficient from the multivariate logistic regression model to a 0- to 100-point scale. Calibration curves, receiver operating characteristic curves, and decision curve analysis (DCA) were used to evaluate the performance of the models across the two cohorts. The optimal cutoff value for the nomogram score was determined using the maximum Youden index. Two-sided *p* values were used, and *p* < 0.05 was considered statistically significant. Data analyzes were performed using SPSS (version 25.0; IBM, Armonk, NY, USA) and R (version 3.6.3; R Foundation for Statistical Computing, Vienna, Austria). Various packages in the R environment were used, including nomogramFormula, rmda, rms, and pROC.

## 3 Results

### 3.1 Clinical characteristics of patients

**Figure 1:**
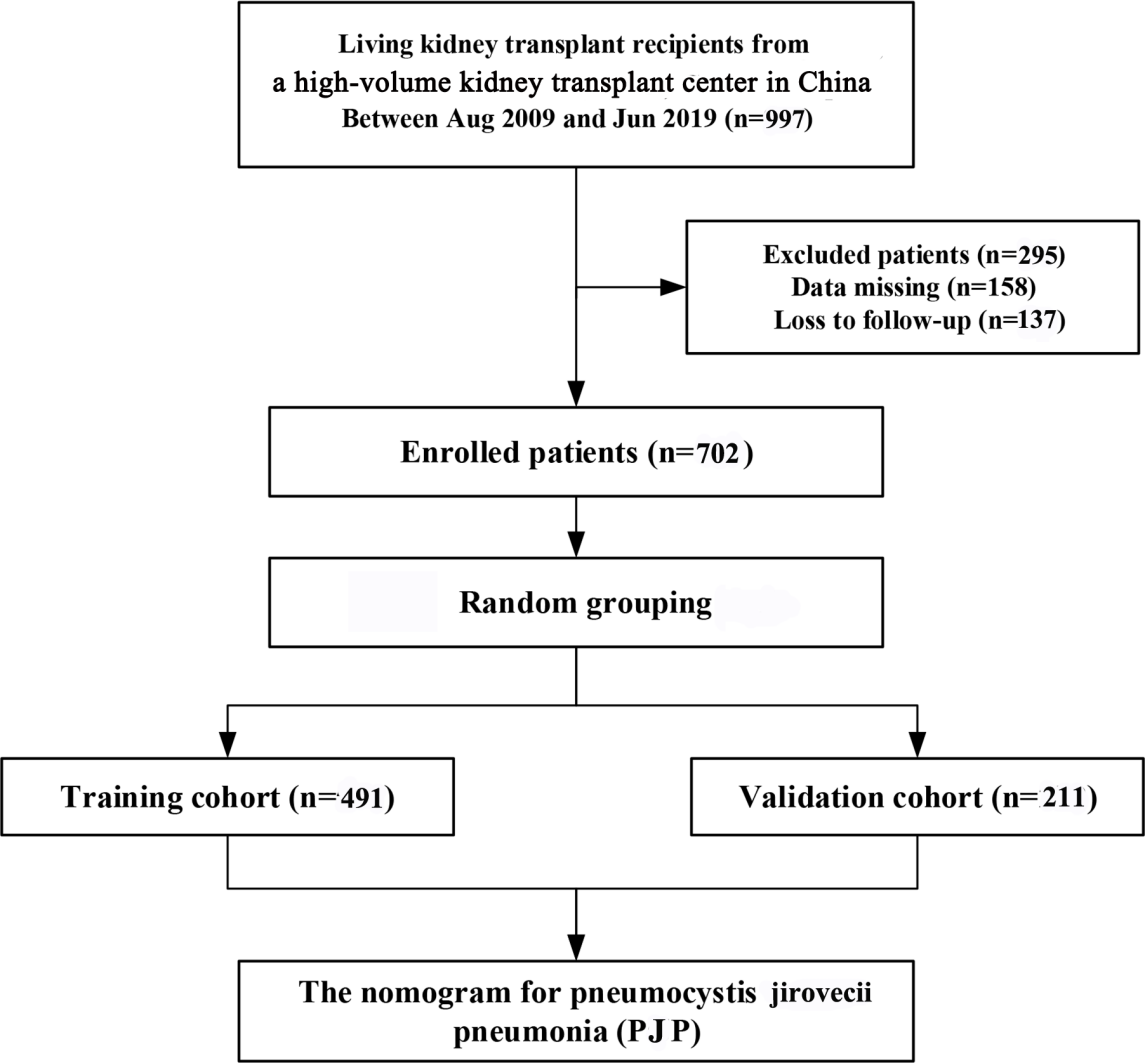
Consort flow diagram for the trial.

During the study period, 997 patients underwent kidney transplantation; 702 met the inclusion and exclusion criteria and were enrolled in this study (**Figure 1**). The median post-transplant follow-up time for the included patients was 17.9 months (IQR: 3.0–35.9 months). A total of 52 patients were diagnosed with PJP after kidney transplantation. The median time from transplantation to PJP diagnosis was 6.2 months (IQR, 2.95-17.79 months). Among these patients, PJP was diagnosed in 1 patient (1.9%) within the first 4 months after transplantation, in 23 patients (44.2%) between 4–6 months, in 26 patients (50.0%) between 6–12 months, and in 2 patients (3.9%) more than 12 months after transplantation. In this study, 4 patients with PJP developed CMV infection after transplantation.

After random allocation, 491 and 211 patients were included in the training and validation cohorts, respectively. A total of 52 patients (7.40%) developed PJP during follow-up, including 38 (7.73%) in the training cohort and 14 (6.64%) in the validation cohort (*p* = 0.61). Compared with the validation cohort, the training cohort had a lower proportion of postoperative antiproliferative agent use [434 (88.4) vs. 200 (94.8), *p* = 0.01]. No statistically significant differences in baseline characteristics or postoperative outcomes were observed between the two cohorts (**Table 1**).

**Table 1:** Baseline characteristics and postoperative outcomes between patients in the training and the validation cohorts (n=702).

Variables	Training cohort (n=491)	Validation cohort (n=211)	*P* value
Age ≤ 60, years	456 (92.9)	201 (95.3)	0.24
Sex, male	352 (71.1)	149 (70.6)	0.77
BMI, kg/m^2^	22.7 ± 3.7	22.9 ± 3.6	0.50
Blood type			
A	142 (28.9)	61 (28.9)	0.92
B	143 (29.1)	61 (28.9)	
AB	167 (34.0)	69 (32.7)	
O	39 (7.9)	20 (9.5)	
Preoperative hemodialysis, N (%)	400 (81.5)	171 (81.0)	0.90
Preoperative ALT	16.2 ± 16.3	17.8 ± 25.3	0.32
Preoperative AST	13.2 ± 13.8	15.1 ± 21.6	0.16
Preoperative HBsAg (+), N (%)	14 (2.9)	10 (4.7)	0.21
Preoperative Anti HCV (+), N (%)	6 (1.2)	2 (0.9)	0.75
Preoperative BK virus infection, N (%)	1 (0.2)	0 (0)	0.51
Preoperative CMV virus infection, N (%)	1 (0.2)	0 (0)	0.51
Preoperative EBV Ab, N (%)	1 (0.2)	1 (0.5)	0.54
Preoperative MOT	18 (3.7)	4 (1.9)	0.22
Induction therapy, N (%)	473 (96.3)	205 (97.2)	0.58
Postoperative antiproliferative agents, N (%)	434 (88.4)	200 (94.8)	**0.01**
Postoperative immunosuppressants, N (%)			0.92
tacrolimus	455 (92.7)	196 (92.9)	
cyclosporine	36 (7.3)	15 (7.1)	
Postoperative tacrolimus, N (%)			0.89
<5 ng/ml	58 (11.8)	25 (11.8)	
5–7 ng/ml	253 (51.5)	110 (52.1)	
≥8 ng/ml	180 (36.7)	76 (36.0)	
Postoperative Sulfonamide treatment, N (%)	315 (64.2)	150 (71.1)	0.08
Postoperative Hb	1218 ± 20.8	119.3 ± 18.8	0.14
Postoperative WBC	7.0 ± 2.9	7.2 ± 3.1	0.51
Postoperative PLT	221.4 ± 67.5	221.0 ± 69.5	0.94
Postoperative NEU percentage (%)	69.47 ± 14.4	71.28 ± 11.0	0.10
Postoperative Cr, μmoI/L	127.40 ± 69.6	123.95 ± 62.3	0.53
Postoperative BUN, mmol/L	9.2 ± 4.9	9.01 ± 4.3	0.69
Postoperative ALT	21.2 ± 14.6	22.05 ± 18.7	0.51
Postoperative AST	17.5 ± 7.8	17.88 ± 8.3	0.55
Postoperative Glu	5.07 ± 3.10	5.79 ± 1.73	0.43
Postoperative CRP, ≥8 μg/ml	93 (18.9)	33 (15.6)	0.30
Postoperative CMV infection,	8 (1.6)	4 (1.9)	0.80
Postoperative Num CD4	550.2 ± 458.8	595.5 ± 472.4	0.24
Postoperative CD4/L(%)	37.9 ± 14.9	38.2 ± 15.7	0.83
Postoperative Num CD8	396.9 ± 320.7	439.6 ± 359.9	0.12
Postoperative CD8/L(%)	29.7 ± 11.0	30.5 ± 11.8	0.38
Postoperative CD4/CD8	1.49 ± 1.00	1.50 ± 1.07	0.96
Patients with PJP	38 (7.7)	14 (6.6)	0.61

Data are presented as n (%) or median (IQR); Bold text hinted that these variables were statistically significant. B, coefficient; OR, odds ratio; BMI, Body Mass Index; ALT, Alanine Aminotransferase; AST, Aspartate Aminotransferase; Hb, hemoglobin; WBC, white blood cell; NEU, neutrophile granulocyte; PLT, Platelet; Glu, Glucose; HCV, Hepatitis C Virus; CMV, Cytomegalovirus; EBV, Epstein-Barr Virus; CRP, C-Reactive Protein; Cr, Creatinine; BUN, Blood Urea Nitrogen; Num CD4, CD4+T cell counts; Num CD8: CD8+T cell counts; L, Lymphocyte. The bold P values indicate statistical significance (p < 0.05).

**Table 2** presents the baseline characteristics of patients with and without PJP. Compared with patients without PJP, kidney transplant recipients with PJP had lower postoperative Cr, CD4, CD8, and CD4/CD8 values [Cr: 56.8 ± 80.4 vs. 123.9 ± 65.7, *p* = 0.01; CD4: 295.3 ± 293.3 vs. 585.3 ± 467.5, *p* < 0.001; CD8: 308.3 ± 384.3 vs. 417.9 ± 327.8, *p* = 0.02; CD4/CD8: 1.2 ± 0.9 vs. 1.5 ± 1.0, *p* = 0.047]. In contrast, the PJP group had a higher rate of preoperative EBV infection and antiproliferative agent use [EBV: 1 (1.9%) vs. 1 (0.2%), *p* = 0.02; antiproliferative agents: 52 (100.0%) vs. 582 (89.5%), *p* = 0.01].

**Table 2:** Baseline characteristics and postoperative outcomes between patients with and without pneumocystis jirovecii pneumonia (n=702).

Variables	Without PJP (n=650)	With PJP (n=52)	*P* value
Age ≤ 60, years	611 (94.0)	46 (88.5)	0.12
Sex, male	464 (71.4)	37 (71.2)	0.97
BMI, kg/m^2^	22.8 ± 3.7	22.1 ± 3.2	0.25
Blood type			0.09
A	190 (29.2)	13 (28.9)	
B	188 (28.9)	16 (29.1)	
AB	222 (34.2)	14 (33.6)	
O	50 (7.7)	9 (8.4)	
Preoperative ALT	16.7 ± 19.8	17.0 ± 14.2	0.92
Preoperative AST	13.9 ± 17.1	12.4 ± 6.6	0.51
Preoperative HBsAg (+), N (%)	23 (3.5)	1 (1.9)	0.54
Preoperative Anti HCV (+), N (%)	8 (1.2)	0 (0)	0.42
Preoperative BK virus infection, N (%)	1 (0.2)	0 (0)	0.78
Preoperative CMV virus infection, N (%)	1 (0.2)	0 (0)	0.78
Preoperative EBV Ab, N (%)	1 (0.2)	1 (1.9)	**0.02**
Preoperative MOT	22 (3.4)	0 (0)	0.18
Induction therapy, N (%)	628 (96.6)	50 (96.2)	0.86
Postoperative antiproliferative agents, N (%)	582 (89.5)	52 (100)	**0.01**
Postoperative immunosuppressants, N (%)			0.22
tacrolimus	605 (93.1)	46 (88.5)	
cyclosporine	45 (6.9)	6 (11.5)	
Postoperative tacrolimus, N (%)			**< 0.001**
<5 ng/ml	68 (10.5)	15 (28.8)	
5–7 ng/ml	334 (51.4)	29 (55.8)	
≥8 ng/ml	248 (38.2)	8 (15.4)	
Postoperative Sulfonamide treatment, N (%)	437 (67.2)	28 (53.8)	0.16
Postoperative Hb	121.3 ± 20.2	117.1 ± 20.5	0.15
Postoperative WBC	7.0 ± 2.9	8.8 ± 3.3	**< 0.001**
Postoperative PLT	220.0 ± 67.9	238.2 ± 68.1	0.06
Postoperative NEU	69.1 ± 13.0	81.0 ± 15.1	**< 0.001**
Postoperative Cr, μmoI/L	123.9 ± 65.7	56.8 ± 80.4	**0.01**
Postoperative BUN, mmol/L	9.0 ± 4.7	10.6 ± 5.1	**0.02**
Postoperative ALT	21.6 ± 15.6	20.0 ± 19.7	0.51
Postoperative AST	17.6 ± 8.0	17.3 ± 7.2	0.79
Postoperative Glu	5.8 ± 2.6	7.8 ± 3.4	**< 0.001**
Postoperative CRP, ≥8 μg/ml	90 (6.9)	36 (34.6)	**< 0.001**
Postoperative CMV infection	8 (1.2)	4 (7.7)	**< 0.001**
Postoperative Num CD4	585.3 ± 467.5	295.3 ± 293.3	**< 0.001**
Postoperative CD4/L	38.1 ± 15.4	36.4 ± 12.0	0.43
Postoperative Num CD8	417.9 ± 327.8	308.3 ± 384.3	**0.02**
Postoperative CD8/L	29.5 ± 11.1	35.7 ± 11.5	**< 0.001**
Postoperative CD4/CD8	1.5 ± 1.0	1.2 ± 0.9	**0.047**

Data are presented as n (%) or median (IQR); Bold text hinted that these variables were statistically significant.

B, coefficient; OR, odds ratio; BMI, Body Mass Index; ALT, Alanine Aminotransferase; AST, Aspartate Aminotransferase; Hb, hemoglobin; WBC, white blood cell; NEU, neutrophile granulocyte; PLT, Platelet; Glu, Glucose; HCV, Hepatitis C Virus; CMV, Cytomegalovirus; EBV, Epstein-Barr Virus; CRP, C-Reactive Protein; Cr, Creatinine; BUN, Blood Urea Nitrogen; Num, Number; L, Lymphocyte. The bold P values indicate statistical significance (p < 0.05).

Postoperative WBC, neutrophil percentage (NEU), blood urea nitrogen (BUN), blood glucose (Glu), C-reactive protein (CRP) (≥8 μg/ml), CMV infection, and CD8/L were higher in the PJP group than in patients without PJP [WBC: 8.8 ± 3.3 vs. 7.0 ± 2.9 10^9^/L, *p* < 0.001; NEU: 81.0 ± 15.1 vs. 69.1 ± 13.0 10^9^/L, *p* < 0.001; BUN: 10.6 ± 5.1 vs. 9.0 ± 4.7 IU/L, *p* = 0.02; Glu: 7.8 ± 3.4 vs. 5.8 ± 2.6 mmol/L, *p* < 0.001; CRP≥8 μg/ml: 36 (34.6%) vs. 90 (6.9%), *p* < 0.001; CMV: 4 (7.7%) vs. 8 (1.2%), *p* < 0.001; CD8/L: 35.7 ± 11.5 vs. 29.5 ± 11.1, *p* < 0.001].

### 3.2 Risk factors and nomogram for PJP

Univariate logistic regression analyzes demonstrated that postoperative NEU (*p* < 0.001), Cr (*p* = 0.002), BUN (*p* = 0.02), Glu (*p* < 0.001), CD4 (*p* < 0.001), CD8 (*p* < 0.001), CD8/L (*p* < 0.001), CD4/CD8 (*p* = 0.04), CRP≥8 (*p* < 0.001), and CMV infection (*p* = 0.002) were risk factors for PJP in kidney transplant recipients (**Table 3**). Multivariate logistic regression analysis showed that postoperative NEU percentage (*p* < 0.001, 95% CI: 1.04-1.12), Glu (p<0.001, 95% CI: 1.11-1.32), CRP≥8 (p<0.001, 95% CI: 4.12-18.29), CD8/L (p=0.01, 95% CI: 1.01-1.09), and CD4 (p=0.02, 95% CI: 0.99-0.99) were independent risk factors for PJP in renal transplant recipients (**Table 3**).

**Table 3:** Univariable and multivariable logistic regression analysis for pneumocystis jirovecii pneumonia.

Variables	Univariable analysis			Multivariable analysis		
	B	OR (95% CI)	*P* value	B	OR (95% CI)	*P* value
Age, > 60 vs. ≤ 60, years	0.74	2.09 (0.84-5.20)	0.11			
Sex, male vs. female	-0.04	0.96 (0.51-1.80)	0.90			
BMI, per kg/m^2^	-0.05	0.95 (0.88-1.03)	0.23			
Blood type						
B vs. A	0.15	1.17 (0.54-2.52)	0.70	0.15	0.86 (0.33-2.30)	0.77
AB vs. A	-0.08	0.92 (0.42-2.01)	0.84	0.29	0.75 (0.29-1.94)	0.55
0 vs. A	0.97	2.63 (1.06-6.50)	**0.04**	0.81	2.25 (0.76-6.69)	0.15
Preoperative ALT, per 10^9^/L	0.00	1.00 (0.99-1.01)	0.87			
Preoperative AST, per 10^9^/L	-0.02	0.98 (0.94-1.03)	0.49			
Hb, per 10^9^/L	-0.01	0.99 (0.97-1.00)	0.09			
Postoperative WBC, per 10^9^/L	-0.00	1.00 (1.00-1.00)	0.84			
Postoperative NEU, per 10^9^/L	0.13	1.13 (1.09-1.18)	**<0.001**	0.08	1.08 (1.04-1.12)	**<0.001**
Postoperative PLT, per 10^9^/L	0.00	1.00 (1.00-1.01)	0.07			
Postoperative ALT, per 10^9^/L	-0.01	0.99 (0.97-1.01)	0.56			
Postoperative AST, per 10^9^/L	-0.01	0.99 (0.96-1.03)	0.89			
Postoperative Glu, per 10^9^/L	0.18	1.20 (1.08-1.33)	**<0.001**	0.19	1.21 (1.11-1.32)	**<0.001**
Preoperative hemodialysis, yes vs. no	-0.19	0.82 (0.41-1.65)	0.58			
Preoperative HBsAg (+), yes vs. no	-0.61	0.55 (0.07-4.12)	0.59			
Preoperative Anti HCV (+), yes vs. no	14.0	0.00 (0.00-Inf)	0.99			
BK virus infection, yes vs. no	12.0	0.00 (0.00-Inf)	0.99			
CMV virus infection, yes vs. no	12.0	0.00 (0.00-Inf)	0.99			
Preoperative EBV Ab, yes vs. no	2.56	12.98 (0.80-210.63)	0.07			
Induction therapy, yes vs. no	-0.15	0.86 (0.20-3.76)	0.84			
Postoperative antiproliferative agents, yes vs. no	16.13	10134172.3 (0.00-Inf)	0.98			
Postoperative CRP≥8 μg/ml, yes vs. no	2.70	14.93 (7.86-28.38)	**<0.001**	2.16	8.68 (4.12-18.29)	**<0.001**
Postoperative CMV infection, yes vs. no	1.92	6.83 (1.98-23.51)	**0.002**	0.87	2.38 (0.35-16.00)	0.37
Postoperative Sulfonamide treatment, yes vs. no	-0.60	0.55 (0.31-0.97)	**0.04**	0.31	1.36 (0.61-3.06)	0.45
Postoperative Cr, per μmoI/L	0.01	1.01 (1.01-1.01)	**0.002**	0.00	1.00 (1.00-1.01)	0.16
Postoperative BUN, mmol/L	0.06	1.06 (1.01-1.11)	**0.02**	0.06	0.94 (0.85 ~ 1.04)	0.25
Postoperative Num_CD4, per unit	-0.01	0.99 (0.99-0.99)	**<0.001**	0.01	0.99 (0.99-0.99)	**0.02**
Postoperative CD4/L	-0.01	0.99 (0.97-1.01)	0.38			
Postoperative Num_CD8	-0.01	0.99 (0.99-0.99)	**0.007**	0.00	1.00 (1.00 ~ 1.00)	0.07
Postoperative CD8/L	0.04	1.05 (1.02-1.07)	**<0.001**	0.04	1.04 (1.01-1.09)	**0.01**
Postoperative CD4/CD8,	-0.40	0.67 (0.45-0.98)	**0.04**	0.30	1.35 (0.94 ~ 1.93)	0.10

Data are presented as n (%) or median (IQR); Bold text hinted that these variables were statistically significant.

B, coefficient; OR, odds ratio; BMI, Body Mass Index; ALT, Alanine Aminotransferase; AST, Aspartate Aminotransferase; Hb, hemoglobin; WBC, white blood cell; NEU, neutrophile granulocyte; PLT, Platelet; Glu, Glucose; HCV, Hepatitis C Virus; CMV, Cytomegalovirus; EBV, Epstein-Barr Virus; CRP, C-Reactive Protein; Cr, Creatinine; BUN, Blood Urea Nitrogen; Num, Number; L, Lymphocyte. The bold P values indicate statistical significance (p < 0.05).

**Figure 2:**
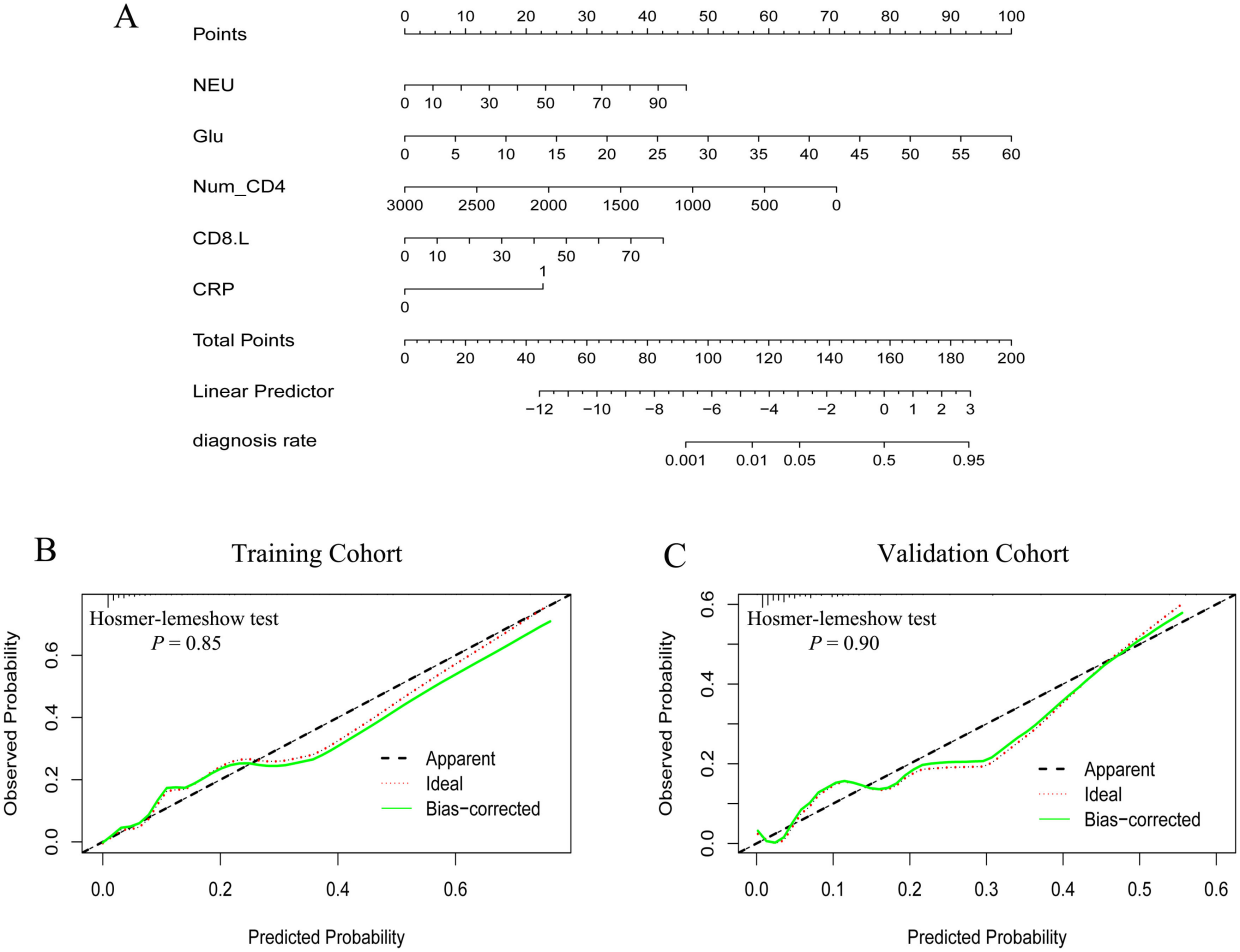
Nomogram for predicting Pneumocystis jirovecii pneumonia (PJP) of kidney transplant recipients and calibration curves in the two cohorts. **(A)** PJP nomogram, the optimal cutoff value of nomogram score was 187, Low-risk group (nomogram score ≤ 187) with the PJP rate no more than 9.0%, and high-risk group (nomogram score > 187) with PJP rate greater than 9.0%; **(B, C)** calibration curves in the training and validation cohorts, respectively.

We then constructed a PJP nomogram to predict PJP in kidney transplant recipients based on these independent prognostic factors (**Figure 2A**). According to the nomogram, the total score and corresponding probability of PJP were calculated for each patient. Based on the maximum Youden index analysis, the optimal cutoff values for the nomogram score and corresponding PJP probability were 109 points and 2.5%, respectively. Patients with a nomogram score and predicted probability of PJP above these cutoff values were classified into the high-risk group, whereas the remaining patients were classified into the low-risk group (**Figure 2A**).

### 3.3 Performance of the PJP nomogram for predicting PJP in the training and validation cohorts

**Figure 3:**
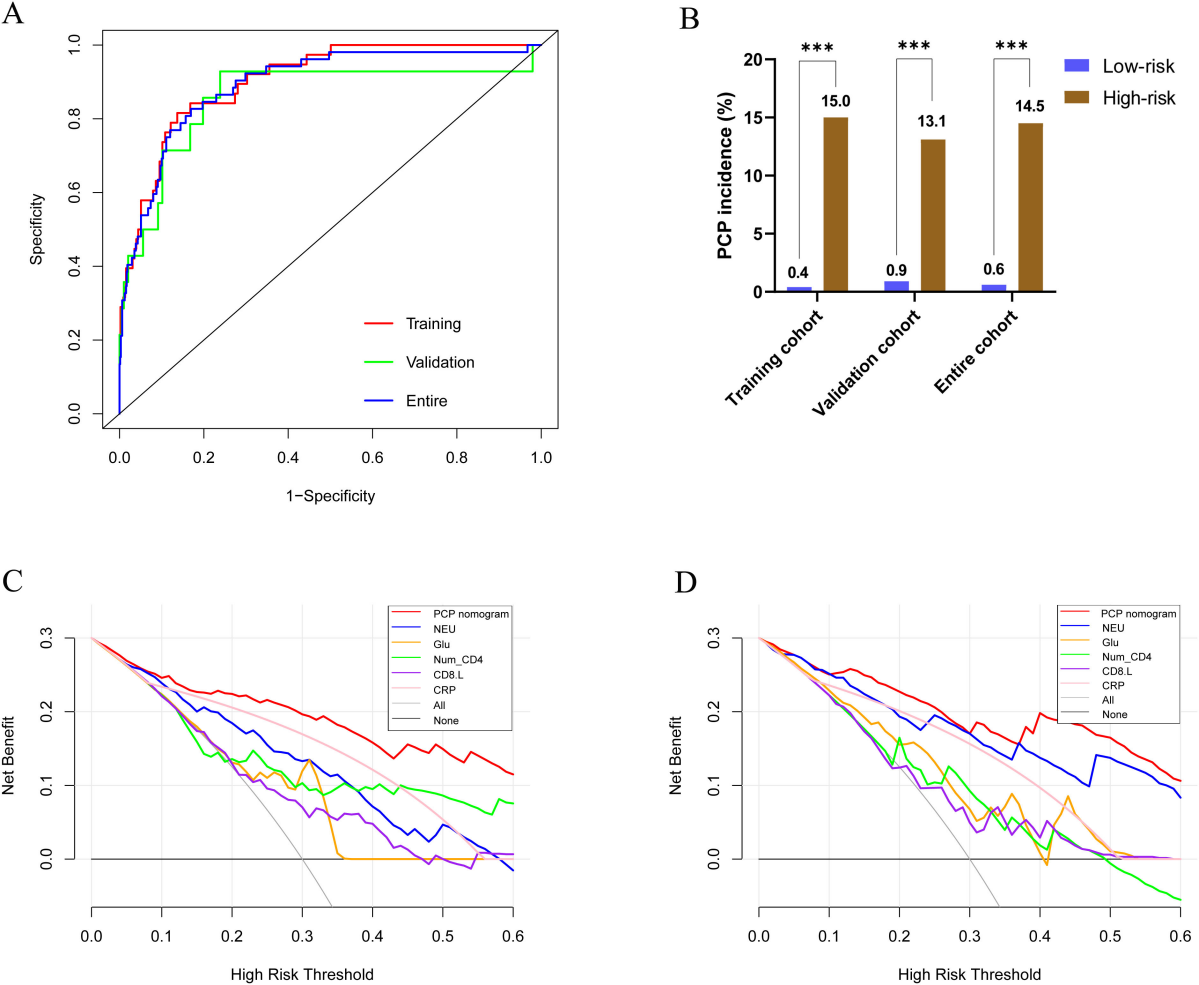
**(A)** The receiver operating characteristic curve analyses of the PJP nomogram in predicting Pneumocystis jirovecii pneumonia (PJP) of the kidney transplant patients in the training and validation cohorts; **(B)** Comparison of PJP rate between patients in low-risk and high-risk groups; **(C, D)** Comparison of net benefits among PJP nomogram and other models in predicting PJP of the kidney transplant patients.

The calibration curves indicated that the PJP nomogram accurately predicted the observed probability of PJP (**Figure 2B, C**; training cohort, *p* = 0.85; validation cohort, *p* = 0.90). The C-statistics or AUC values of the nomogram for PJP prediction were 0.88 (95% CI, 0.83-0.94), 0.93 (95% CI, 0.87-0.98), and 0.89 (95% CI, 0.85-0.94) in the training, validation, and entire cohorts, respectively (**Figure 3A**).

The discriminative performance of the nomogram was further evaluated by dividing the predicted probability of PJP into two risk groups based on the nomogram score (low-risk group, nomogram score ≤ 109; high-risk group, nomogram score > 109). The incidence of PJP was significantly higher in the high-risk group than in the low-risk group across all three cohorts (**Figure 3B**; training cohort: 15.0% vs. 0.4%; validation cohort: 13.1% vs. 0.9%; entire cohort: 14.5% vs. 0.6%, all *p* < 0.001).

### 3.4 Comparison of the predictive power of clinical net benefit between the PJP nomogram and other models

DCA transforms complex mathematical models into simple, easy-to-interpret graphics, allowing intuitive assessment of the clinical utility and net benefit of different models [18]. **Figure 3C, D** shows that the PJP nomogram provided greater net benefit in both the training and validation cohorts than the other four models based on independent prognostic factors.

## 4 Discussion

In this study, based on nomogram analysis and other methods, we identified specific differences in 702 patients after kidney transplantation between those with and without PJP. Lower postoperative CD4 T cell count and higher CD8/L, blood glucose, NEU percentage, and CRP levels were all independent risk factors for the development of PJP in renal transplant recipients. Furthermore, a novel nomogram was developed to predict PJP development. To our knowledge, this is the first study to develop a prognostic risk profile model for PJP based on independent risk factors and stratified nomogram scores for low-risk (≤ 109) and high-risk (> 109) PJP incidence in a large sample of renal transplant recipients. In addition, this study demonstrated that the PJP nomogram showed good discrimination (C-index of 0.88) and provided greater net benefit in both the training and validation cohorts than models based solely on individual prognostic factors. Thus, these findings may help improve the prediction of PJP risk and inform an optimal individualized prophylaxis strategy for renal transplant recipients.

Lymphocytes are recognized to play a critical role in the development of PJP in immunocompromised patients. Among them, T cells are crucial for host defense against Pneumocystis (Iriart et al., 2015; Cao et al., 2022). They are essential for pathogen clearance; however, quantitative deficiency, functional impairment, or subset imbalance can lead to either pathogen evasion or excessive and inappropriate inflammatory responses that result in severe lung injury. Therefore, the pathogenesis of PJP depends on the complex dynamic equilibrium among different T-cell subsets and between T cells and the pulmonary microenvironment (Chen et al., 2025). Distinct subsets of CD4+ and CD8+ T cells have been shown to participate in PJP development through specific cytokines and interactions with other immune cells, thereby significantly influencing pulmonary fungal burden and disease severity (Li et al., 2017; Messiaen et al., 2017; Liu et al., 2020; Cheng et al., 2024; Chen et al., 2025). However, the predictive value of CD4^+^ and CD8^+^ T-lymphocyte markers for PJP risk in renal transplant recipients remains unclear. In this study, univariate logistic regression analysis revealed significant associations between PJP and several postoperative lymphocyte markers, including CD4+ T-cell count, CD8+ T-cell count, CD8/L, and CD4/CD8, while multivariate analysis identified only lower CD4^+^ T-cell counts and higher CD8/L as independent risk factors for PJP. Furthermore, these two factors were subsequently incorporated into a nomogram model, demonstrating their predictive value for PJP infection. These findings align with and extend previous evidence. A study by Chen et al. identified reduced TCR diversity in CD4^+^ T cells. However, it increased diversity in CD8^+^ T cells, highlighting the critical role of T cells in orchestrating the immune response to PJP infection (Chen et al., 2025). CD4^+^ T cells play a central role in immune defense against PJP, and their functional status ultimately determines the course of infection and outcome (Thomas and Limper, 2004; De la Rua et al., 2016; Kaminski et al., 2021). Immunodeficiency models research showed that CD4+ T-cell loss leads to PJP susceptibility, and their transfer into Rag1-/- mice clears the infection (Opata et al., 2015). Thus, CD4+ T-cells are central to host defense against this pathogen. In people with HIV, loss of CD4^+^ T cells increases susceptibility to PJP, and the degree of CD4^+^ lymphopenia correlates with risk of PJP development and mortality (Freiwald et al., 2020; Douvali et al., 2025). Similarly, lower CD4^+^ T cell counts or CD4+ T cell percentages have been reported to be associated with PJP risk and worse outcomes in renal transplant recipients (Schürmann et al., 2013, Iriart et al., 2015; Freiwald et al., 2020; Cheng et al., 2024). Our results showed that CD4(+) T cell counts differed significantly between the PJP and non-PJP groups, and in further validating the predictive value of lower CD4^+^ T cell counts for PJP risk. In addition, there is stronger evidence that CD8+ T cells are required for the development of respiratory impairment in PJP, although the mechanisms of CD8+ T-cell-mediated damage remain unclear (De la Rua et al., 2016; Cao et al., 2022). Previous studies have observed elevated CD8^+^ lymphocyte levels in infected immunocompromised patients, and an effective CD8^+^ T-cell response may influence disease severity (Walzer et al., 2008; Li et al., 2020; Ali et al., 2022; Liu et al., 2020), and there is a positive factor for response to Pneumocystis jirovecii during the depletion of CD4+ T-cells (Fishman and Gans, 2019; Zhu et al., 2024). In a model of PJP infection, CD8 T cells emerged as critical to the pathophysiology of lung injury, alongside CD4 T cells (Swain et al., 2006). Therefore, it is reasonable to speculate that CD8(+) T cells could also be an important predictor of PJP. However, the predictive role of CD8+ lymphocytes for PJP risk in renal transplant recipients requires further validation. Collectively, these findings indicate that CD4^+^ T cell count and CD8/L serve as valuable clinical markers for assessing the risk of PJP in renal transplant recipients, thereby highlighting the importance of immune imbalance in determining susceptibility to infection.

A novel finding of this study is that elevated postoperative serum NEU percentage and CRP levels, as inflammatory markers, are independent risk factors for PJP in renal transplant recipients. In our cohort, both markers were significantly higher in patients who developed PJP than in non-PJP controls. The role of inflammatory markers in PJP has attracted growing research interest. Previous studies have indicated that NEU percentage in BALF may be a prognostic marker of poor outcome in patients with PJP (Lee et al., 2015). Similarly, the peripheral blood neutrophil-to-lymphocyte ratio has been shown to have predictive value for PJP occurrence and severity in non-HIV populations (Li et al., 2023; Wang et al., 2025). As a major acute-phase protein, CRP has also been reported to be independently associated with PJP development, disease severity and poor outcome (Chung et al., 2022; Gao et al., 2024; Zheng et al., 2025). However, specific data on the predictive roles of NEU percentage and CRP for PJP in renal transplant recipients remain limited; therefore, further validation studies are needed. From our findings, we hypothesize that an early hyperinflammatory response triggered by postoperative infection may initiate a state of inflammation-related immune dysregulation, culminating in long-term immune exhaustion. This persistent immune imbalance can impair T-cell reconstitution (resulting in lymphopenia), thereby creating a permissive environment for the reactivation of Pneumocystis jirovecii. This suggest that postoperative inflammatory markers may reflect a persistently disordered immune system.

Metabolic dysfunction is known to promote immune dysregulation and increase infection risk (Shi et al., 2024; Alessa et al., 2025; Wang et al., 2025). A key manifestation, hyperglycemia, impairs the defense capacity of immune cells and disrupts immune homeostasis (Kim and Choi, 2025). However, its specific role in predisposing patients to PJP remains poorly defined. Hosseini-Moghaddam et al. found that diabetes was significantly more prevalent among solid organ transplant recipients with PJP (Hosseini-Moghaddam et al., 2019). Similarly, Leth et al. identified hyperglycemia as a potential contributing risk factor for PJP in transplant recipients (Leth et al., 2014). However, conclusive evidence identifying hyperglycemia as a significant independent risk factor for PJP remains scarce. Our findings build upon and extend these observations by demonstrating a statistically significant association between postoperative hyperglycemia and PJP risk in renal transplant recipients. Collectively, these consistent results suggest that diabetes contributes substantially to PJP risk and that elevated postoperative blood glucose may serve as a practical predictor of PJP after kidney transplantation, offering a potential target for risk stratification in recipients who require more attention.

It has been reported that CMV infection is associated with the development of PJP after kidney transplantation, presumably due to excessive immunosuppression (Garg et al., 2018; Hosseini-Moghaddam et al., 2019). Furthermore, extended prophylaxis for PJP has been recommended after CMV infection. However, in this study, postoperative CMV infection was associated with PJP risk only in univariate analysis, not in multivariate analysis, possibly because of the low incidence of CMV infection with routine antiviral prophylaxis after transplantation. Additionally, no significant association was observed between induction therapy and risk of PJP.

Notably, most PJP cases in this study occurred after completion of the standard 3-4-month postoperative prophylaxis, and no breakthrough infections were observed. The incidence of PJP was 7.4% (52/702) during follow-up, and the vast majority of PJP cases occurred within 12 months after kidney transplantation in this study. Compared with other reports, this infection rate is relatively high, possibly because of insufficient prophylactic sulfonamide treatment for 3–4 months after transplantation at our center. Thus, we suggest continuing PJP prophylaxis for ≥6–12 months post-transplantation in renal recipients at risk of PJP.

This study had several limitations. First, its retrospective design inevitably introduced selection bias. Owing to the low incidence of PJP and limited event counts, randomized prospective trials are difficult to conduct; therefore, most studies on PJP in organ transplantation have used a retrospective case-control or cohort design. Second, all patients in this study were enrolled at a single center and lacked external validation. To the best of our knowledge, this is the largest study to investigate risk factors for PJP and to develop a nomogram for PJP in renal transplant recipients. In the future, we will initiate a prospective and multicenter study to validate the nomogram for PJP after renal transplantation.

## 5 Conclusion

PJP is a severe opportunistic fungal infection in which immune responses to Pneumocystis may involve complex interactions among T lymphocytes, inflammatory neutrophils, and metabolic abnormalities. Renal transplant recipients with lower CD4 T cell counts and higher CD8/L, blood glucose, NEU percentage, and CRP levels have an increased risk of PJP. Our nomogram model may be a useful, convenient, and applicable tool for predicting the individualized risk of PJP after kidney transplantation. This model may enable risk stratification to guide tailored prophylaxis, extending it for high-risk patients. Multicenter prospective validation, recalibration, and clinical utility testing are necessary before the model can be incorporated into post-transplant prophylaxis algorithms.

## Data Availability

The raw data supporting the conclusions of this article will be made available by the authors, without undue reservation.
